# Lightweight, Compact, and High-Sensitivity Passive Fourier Transform Infrared Spectroscopy-Based Gas Detection System

**DOI:** 10.3390/s26051493

**Published:** 2026-02-27

**Authors:** Xiangning Lu, Min Huang, Wenbin Ge, Lulu Qian, Zhanchao Wang, Yan Sun, Jinlin Chen, Wei Han

**Affiliations:** 1 Aerospace Information Research Institute, Chinese Academy of Sciences, Beijing 100094, China; luxn@aircas.ac.cn (X.L.);; 2 State Key Laboratory of Remote Sensing and Digital Earth, Beijing 100094, China; 3 School of Optoelectronics, University of Chinese Academy of Sciences, Beijing 100049, China; 4 AVIC Guizhou Aircraft Co., Ltd., Anshun 561000, China

**Keywords:** fourier transform infrared spectroscopy, gas detection, real-time monitoring, high precision data acquisition

## Abstract

With the intensification of environmental pollution and the increasingly prominent problem of industrial harmful gas emissions, existing mainstream gas detection technologies still have obvious limitations in terms of real-time performance, non-contact capability, detection accuracy, and multi-component identification. To address this demand, this paper proposes a lightweight and compact gas detection system based on passive Fourier Transform Infrared Spectroscopy (FTIR). The system innovatively integrates an improved parallel pendulum mirror interferometer and a low-noise signal preprocessing module, and simultaneously presents a novel oversampling method fusing equal time, equal optical path difference, and digital filtering, which effectively enhances the operational stability and sampling accuracy of the spectrometer. The system features excellent platform adaptability and can be flexibly mounted on various operation carriers. Combined with a two-dimensional rotating platform and an inertial navigation module, its monitoring range and application scenarios can be further expanded. Indoor sensitivity test results show that the detection limit of the system for sulfur hexafluoride (SF_6_) is less than 20 ppm; flight tests under real-world scenarios have successfully achieved accurate detection of SF_6_ gas, fully verifying the practical application effectiveness of the system. Based on the comprehensive results of indoor and outdoor tests, the system demonstrates core technical advantages of high sensitivity, strong flexibility, and excellent real-time performance. It is expected to be widely applied in gas monitoring tasks across multiple fields such as industrial safety monitoring, ecological environment monitoring, and transportation support in the future.

## 1. Introduction

In contemporary society, air pollution and hazardous gas leakage have emerged as pressing global challenges, necessitating reliable gas monitoring across diverse fields including industrial safety, environmental surveillance, and transportation operations. Currently, mainstream gas detection technologies encompass electrochemical sensors, semiconductor sensors, Tunable Diode Laser Absorption Spectroscopy (TDLAS), and Gas Chromatography/Gas Chromatography-Mass Spectrometry (GC/GC-MS), among others. However, these methods suffer from inherent limitations: some are confined to single-component detection, others exhibit prolonged response times incompatible with online real-time monitoring, and many lack robust anti-interference capabilities. Consequently, they fail to meet the growing demand for a comprehensive gas monitoring solution that integrates real-time performance, online operation, non-contact measurement, high precision, and multi-component analysis [1,2,3,4].

Fourier Transform Infrared Spectroscopy (FTIR) leverages the selective absorption of infrared radiation by gas molecules. When irradiated with infrared light, gas molecules absorb photons of specific wavelengths, triggering transitions in their internal vibrational and rotational energy levels. Due to structural and chemical bond differences, distinct gas molecules exhibit unique absorption spectra—analogous to “spectral fingerprints”—enabling their identification. By applying Fourier transform algorithms to convert detected interferograms into spectra, FTIR enables accurate detection and quantification of greenhouse gases, toxic substances, and flammable/explosive gases based on these characteristic spectral signatures [5,6,7,8,9].

As an advanced infrared spectral acquisition technique, FTIR operates on the principle of interferometry, boasting inherent advantages such as high optical throughput, multi-channel detection, superior resolution, high signal-to-noise ratio (SNR), broad spectral coverage, and rapid scanning speeds. These attributes make it a powerful tool for spectral analysis, capable of measuring wide-range, complex, or ultra-weak spectra [10,11,12,13].

Passive Fourier Transform Infrared spectrometers do not require a dedicated infrared light source, but instead utilize ambient radiation as the infrared radiation source. By selecting infrared-transmissive window lenses compatible with the corresponding spectral range, they capture the infrared characteristic information of target gases and conduct material species analysis. This non-contact monitoring approach enables remote measurement without gas sampling, thereby avoiding safety hazards associated with direct exposure to toxic/harmful gases and minimizing potential errors introduced during sampling.

The commercial FTIR spectrometers suffer from inherent drawbacks of bulkiness, heavy weight and short detection range. Such structural and performance limitations make the commercial FTIR spectrometers lack the adaptability for multi-platform integration, especially for airborne and vehicle-mounted applications. When engineered as a lightweight, compact system, passive FTIR spectrometers can be seamlessly integrated into various platforms—including automobiles, unmanned aerial vehicles (UAVs), and aerostats—offering exceptional flexibility for expanded application scenarios. Such systems provide comprehensive, high-fidelity data support for pollution control, safety management, military reconnaissance, and other critical fields [14,15,16].

In view of the drawbacks of the aforementioned gas detection technologies, and taking advantage of the merits of passive FTIR, this paper designs a lightweight and compact gas detection system based on passive Fourier transform infrared spectroscopy technology. The system enables remote control and telemetry via wireless networks, and is capable of non-contact, high-sensitivity, real-time and high-efficiency gas detection. Section 2 details the overall system design, encompassing its composition, optical configuration, electronic control system, mechanical structure of the FTIR module, spectral reconstruction principle and gas identification method. Section 3 describes indoor gas sensitivity tests and UAV-borne flight tests for gas detection. Finally, the paper concludes with a discussion of the system’s performance and key findings.

## 2. Gas Detection System Methods

### 2.1. System Composition and Application Scenarios

**Figure 1:**
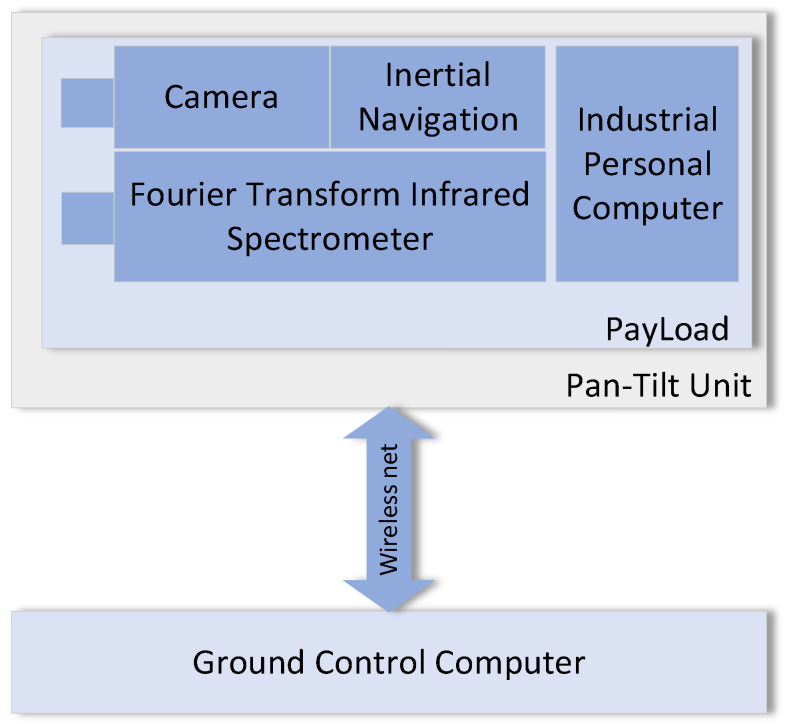
System composition.

The proposed gas detection system comprises a passive Fourier transform infrared (FTIR) spectrometer, a two-dimensional pan-tilt unit, a visible light camera, an inertial navigation module (INM), a micro industrial computer, a wireless network transmission module, and a ground control computer. The system composition is illustrated in Figure 1.

**Figure 2:**
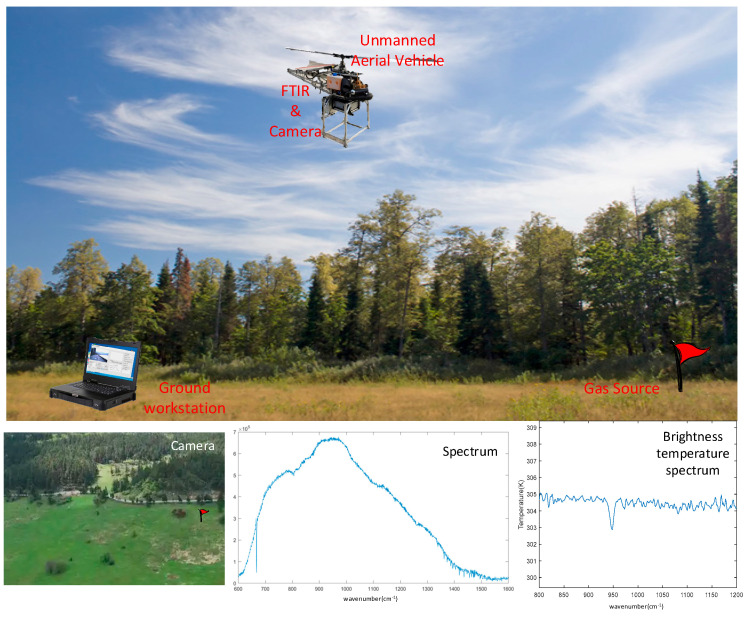
Application scenario.

Operating platforms can be flexibly selected to match specific detection requirements, including ground tripods, automobiles, aerostats, or unmanned aerial vehicles (UAVs). A representative application scenario with a UAV as the deployment carrier is illustrated in Figure 2.

In the aforementioned scenario, a UAV serves as the payload-carrying platform. The workflow is outlined as follows:

1. Prior to the platform’s takeoff, a 28VDC power supply is activated, initiating the cooling process of the FTIR detector’s cooler. Simultaneously, the pan-tilt unit, visible light camera, INM, micro industrial computer, and wireless network transmission module are powered on and enter operational mode. During this phase, the ground workstation establishes a connection with the micro industrial computer integrated within the spectrometer via the wireless network to configure the FTIR’s operating parameters.
2. Once the detector cooling process is completed, the UAV takes off. Upon reaching the preset altitude, the UAV hovers to collect background spectral data in the vicinity of the target area. Based on the camera imagery transmitted to the ground workstation, the platform or pan-tilt attitude is adjusted to align the target within the FTIR’s field of view, enabling the acquisition of raw target spectral data.
3. On the ground workstation, the collected raw target and background spectral curves are converted into brightness temperature spectra. Utilizing a target recognition algorithm, the system detects the presence of the target gas and identifies its type. Concurrently, the latitude and longitude coordinates of the target location are determined using inertial navigation data. The key parameters of the gas detection system are summarized in Table 1.

### 2.2. Infrared Spectrometer Optical System

**Figure 3:**
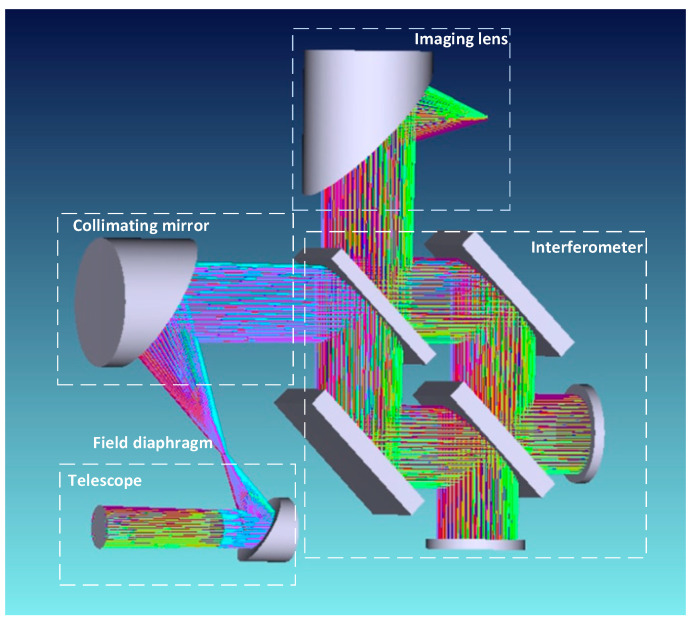
Three-dimensional structural diagram of the optical system.

The optical system of the FTIR spectrometer primarily consists of a fore optic, a collimating mirror, an interferometer, and an imaging mirror. The three-dimensional (3D) structural diagram of the optical system is illustrated in Figure 3. The fore optic collects infrared radiation emitted by the target and converges it onto the field stop. The light beam exiting the field stop passes through the collimating mirror and enters the interferometer, where it is split into two coherent beams by the beam splitter. These two beams are reflected by the moving mirror and fixed mirror, respectively, then propagate back along their original paths to recombine and generate interference. Finally, the interference light is focused onto the detector via the imaging mirror, enabling the acquisition of interference signals.

**Figure 4:**
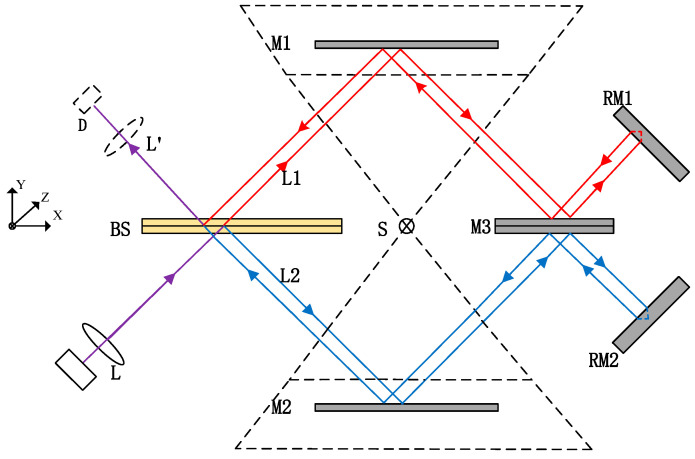
Interferometer optical path diagram.

The interferometer within the optical system serves as the optical core of the entire Fourier transform spectrometer. This system adopts an improved Michelson interferometer configuration—specifically a parallel pendulum mirror interferometer—characterized by high stability and robust anti-interference capability. Its operational principle is illustrated in Figure 4, comprising key components: a beam splitter (BS), three plane mirrors (M1, M2, M3), two end mirrors (RM1, RM2), and a moving mirror motion mechanism. Notably, both upper and lower surfaces of mirror M3 are reflective; the reflective surfaces of M1, M2, and M3 are mutually parallel, with M1 and M2 symmetrically arranged relative to M3. These three mirrors are fixedly integrated to form a parallel pendulum mirror assembly—the sole moving component of the interferometer—which can oscillate around a rotation axis S perpendicular to the paper plane (Z-direction). The two end mirrors, RM1 and RM2 are oriented perpendicular to the incident light direction.

The interference signal acquisition process proceeds as follows: Parallel light carrying target measurement information enters the interferometer, where the beam splitter (BS) splits it into two beams: transmitted light and reflected light. The transmitted light is reflected sequentially by mirrors M1 and M3 before irradiating end mirror RM1, and then returns to BS after reflection by RM1. Similarly, the reflected light is reflected successively by mirrors M2 and M3, incidents on end mirror RM2, and subsequently returns to BS following reflection by RM2. The two beams converge at BS to form coherent light, which is focused by the imaging mirror onto detector D, enabling the acquisition of the target’s interference intensity. As the parallel pendulum mirror assembly oscillates, the two light beams passing through the interferometer generate a time-varying optical path difference (OPD), thereby allowing the detector to collect target interference information that varies with OPD. After recording the complete interference data of the target, the original spectral information can be retrieved via spectral reconstruction algorithms.

The OPD of the system varies with the oscillation of the parallel pendulum mirror assembly. Let the distance between the parallel reflective surfaces be h, the incident angle of light on the parallel pendulum mirror be θ, and the swing angle of the pendulum mirror be α. When the assembly is in the central position, the incident angle of light on mirrors M1 and M2 is 45°, and the system’s optical path difference is expressed as:

(1) Δ = 2 h [cos (θ − α) − cos (θ + α)]

**Figure 5:**
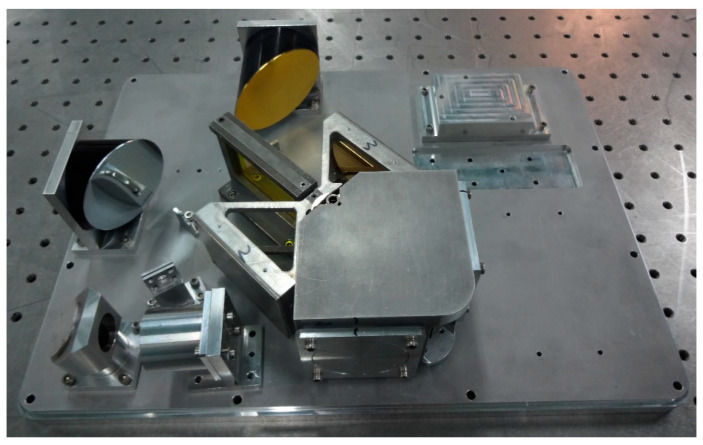
FTIR optical path alignment diagram.

This system features a simplified OPD model. The optical path difference can be adjusted by modifying the distance between the two parallel reflectors *h* and the swing amplitude of the pendulum mirror α, thereby maximizing the spectrometer’s spectral resolution within a limited spatial envelope. The actual optical path layout is illustrated in Figure 5.

### 2.3. Electronic Control System of the Infrared Spectrometer

**Figure 6:**
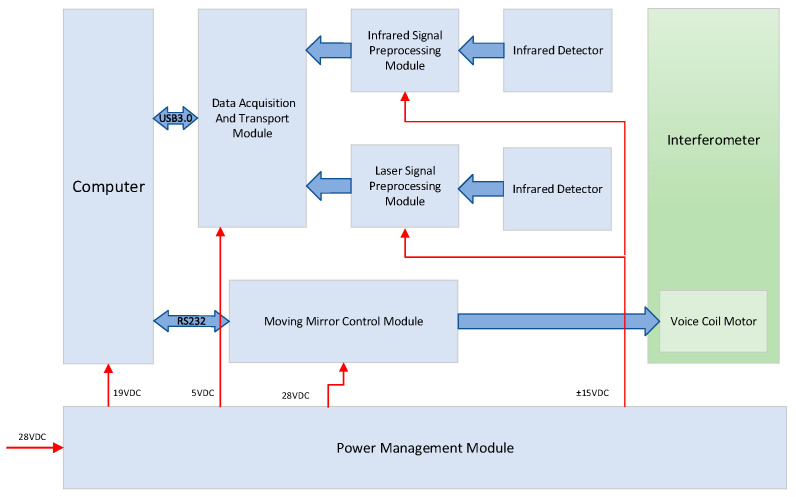
Composition of the electronic control system.

The electronic control system of the FTIR spectrometer is primarily responsible for interference signal processing, acquisition, and transmission, as well as the control of the parallel pendulum mirror. It is mainly composed of a data preprocessing module, a data acquisition and transmission module, a pendulum mirror control module, and a power management module. The electronic control system as shown in Figure 6.

The infrared interference signal generated by the optical system from the target infrared radiation source is detected by the infrared detector, and the output signal is then transmitted to the infrared signal preprocessing module to be conditioned into an interference signal compatible with the voltage range of the data acquisition module. Meanwhile, a reference laser beam also passes through the interferometer to produce a laser interference signal, which is adjusted to an appropriate voltage range via the laser signal preprocessing module. The data acquisition and transmission module synchronously collects the infrared and laser interference signals and transmits them to the micro industrial computer through a USB 3.0 interface. On the computer, the zero-crossing points of the laser interference signal are utilized to resample the infrared interference signal with equal OPD. Subsequently, the infrared interference signal resampled at equal OPD undergoes Fast Fourier Transform (FFT) to ultimately generate the target spectral curve. Additionally, the laser interference signal can synchronously reflect the displacement of the parallel pendulum mirror, and the position information is fed back to the pendulum mirror control module to regulate the uniform oscillation of the interferometer’s pendulum mirror.

#### 2.3.1. Signal Preprocessing Module

The signal preprocessing module comprises an infrared signal amplification circuit, a laser signal amplification circuit, and an analog signal filtering and conditioning circuit. For infrared detection, a Stirling-cooled mercury-cadmium-telluride (MCT) photoconductive infrared detector is adopted. This detector features a broad spectral range, rapid response speed, and high detectivity for high-frequency signal reception, rendering it well-suited for real-time online monitoring scenarios. Its structural diagram is illustrated in Figure 6, with detailed specifications summarized in Table 2.

Since the photoconductive MCT detector outputs a microampere-level current signal, the system is equipped with a low-noise, high-bandwidth, and high-gain signal amplification circuit. For the constant current source bias circuit, a low-power NPN transistor with excellent current characteristics and high gain is selected, operating in the amplification region to provide a precisely adjustable bias current. The primary amplification stage employs a closed-loop feedback T-type transimpedance amplifier circuit, which enables low-noise current-to-voltage (I–V) conversion and amplification of weak current signals while minimizing the impact of offset voltage and thermal noise on the weak signals. The secondary amplification stage adopts an operational amplifier-based negative feedback configuration to further boost signal gain. The gain of the amplifying circuit can reach 1000, with a noise level of less than 1 nV/Hz^1/2^ and a bandwidth ranging from 1 Hz to 200 kHz.

**Figure 7:**
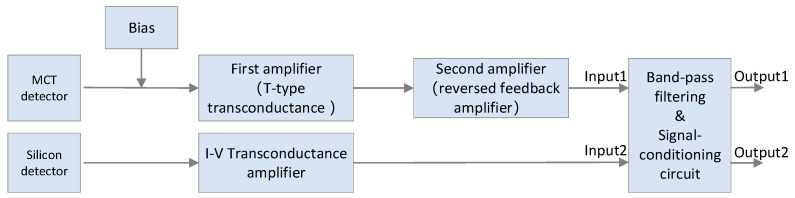
Signal preprocessing module schematic diagram.

For the laser detection path, a silicon photodiode is utilized, with its signal amplification circuit implementing a single-stage transimpedance I–V amplifier. After amplification, both the infrared and laser signals pass through a 100 Hz–200 kHz band-pass filter circuit to eliminate DC components and high-/low-frequency noise. Subsequently, the signals are fed into a subtractor circuit to achieve DC bias adjustment, conditioning the infrared and laser interference signals to match the voltage range of the data acquisition module. The circuit schematic is illustrated in Figure 7.

#### 2.3.2. Signal Acquisition and Transmission Module

Traditional signal acquisition methods impose strict requirements on the phase of sampling points when performing equidistant sampling of interference signals. However, factors such as pendulum mirror jitter, analog filter distortion, inter-channel sampling delay, and circuit random noise inevitably introduce various types of errors into interference signal sampling. These errors degrade the infrared spectrum inversion accuracy and may lead to misjudgment. To address this issue, the FTIR system employs a data sampling method for infrared interferograms that combines equal-time sampling and OPD sampling. This integrated acquisition approach mitigates signal intensity and phase distortion caused by circuit imperfections and reduces the impact of sampling errors on spectral reconstruction [17,18,19,20,21].

**Figure 8:**
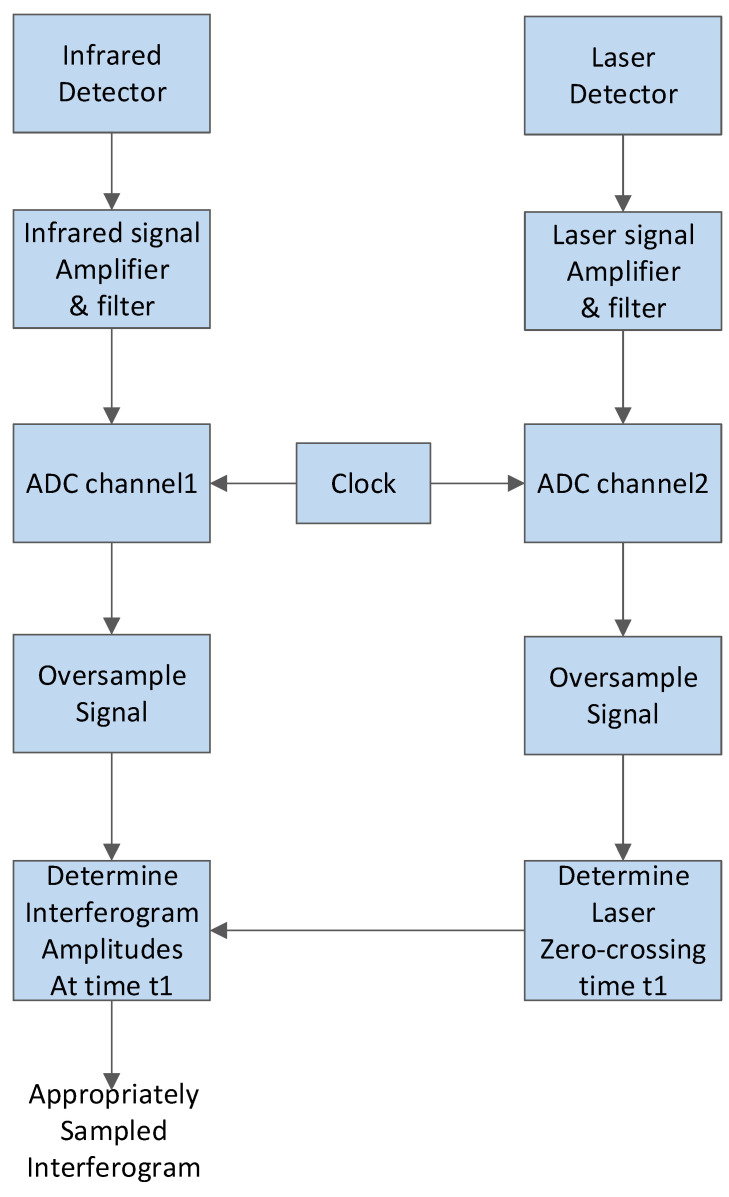
Data acquisition flow chart.

The signal acquisition and transmission module adopts a dual-channel synchronous sampling analog-to-digital converter (ADC) to perform equidistant time-interval oversampling on infrared and reference laser signals simultaneously. The raw infrared and laser interference signals are then transmitted to a computer, where digital filtering algorithms are applied to optimize the interference signals and identify the zero-crossing points of the reference laser signal. By re-sampling the infrared interference signal using the zero-crossing moments as trigger points, an infrared interference signal sampled at equal optical path differences is obtained. The sampling process is illustrated in Figure 8.

**Figure 9:**
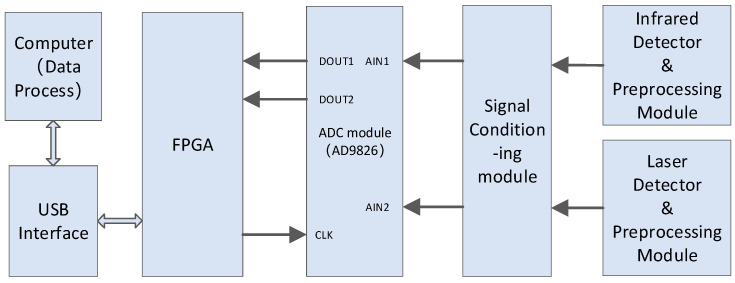
Composition block diagram of the data acquisition system.

The data acquisition module consists of an ADC circuit, an FPGA main control circuit, and a USB communication interface circuit. Firstly, the signal amplitudes of the infrared interference signal and the laser interference signal are conditioned to match to the voltage range of the ADC input, and then analog-to-digital conversion is performed through the two channels of the ADC at the same time. The FPGA provides sampling control timing and data buffer space for the ADC, and finally, the data is transmitted to the host computer through the USB 3.0 interface. The data is digitally filtered and spectrally restored by the processing software in the host computer. The principle block diagram is as Figure 9.

The AD chip selects the high-bandwidth, low-noise AD9826 chip with 16-bit sampling resolution, which supports dual-channel synchronous acquisition and a maximum sampling frequency of 4MPS. The USB3.0 interface chip selects FT601Q, which acts as a conversion from USB3.0 PHY to FIFO. The collected data is written into FT601 and transmitted to the PC.

#### 2.3.3. Power Management Module

**Figure 10:**
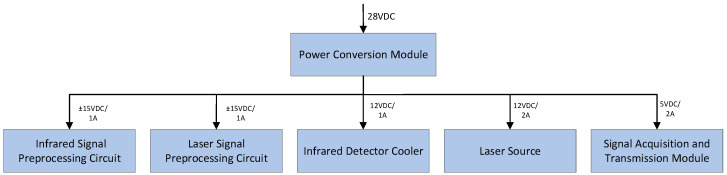
Schematic diagram of voltage conversion.

The FTIR system draws external power from the operating platform, which provides a 28 VDC supply. The power management module converts this input voltage into multiple regulated output channels: two sets of ±15 VDC for the infrared signal preprocessing circuit and laser signal preprocessing circuit, respectively; two 12 VDC supplies dedicated to the infrared detector cooler and reference laser source; and a 5 VDC output for the signal acquisition and transmission module. The physical implementation of the power management module is illustrated in Figure 10.

### 2.4. Infrared Spectrometer Mechanical Structure

**Figure 11:**
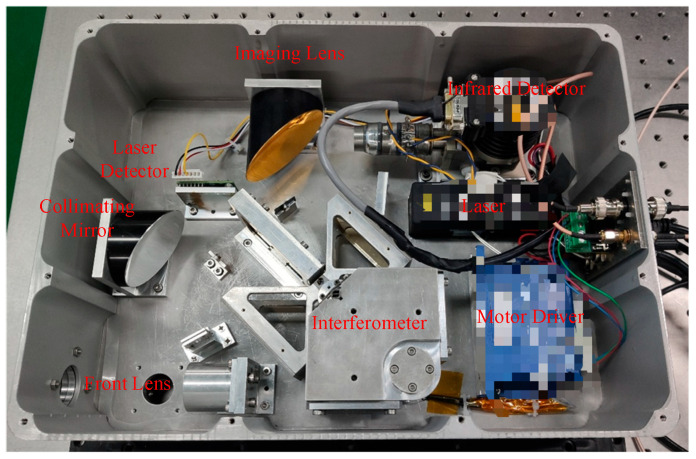
The structure of the spectrometer.

Based on the aforementioned optical system design, the mechanical structure of the FTIR spectrometer is illustrated in Figure 11. On the premise of ensuring the positional accuracy of optical components, aluminum alloy is adopted as the primary material to control the overall weight of the system. The front end is equipped with an 8–14 μm infrared window to filter out stray light of other wavelength bands, while the rear end is connected to the electronic control box via detector signal lines, power cables, and motor control cables. The dimensions of the main body are 340 mm × 250 mm × 140 mm, with a total weight of 8.7 kg.

The spectrometer main body integrates key components including a fore optic, collimating mirror, interferometer, imaging mirror, infrared detector, reference laser, and laser detector. Notably, the fore optic, collimating mirror, and imaging mirror are all off-axis parabolic mirrors with gold-plated surfaces, ensuring excellent reflectivity across the wide spectral range of the long-wave infrared (LWIR) band (8–14 μm). The interferometer comprises a beam splitter assembly, a parallel pendulum mirror assembly, two end mirrors, and a voice coil motor (VCM). The pendulum mirror assembly is connected to the base via a pair of torsion springs, enabling oscillation within a specified angular range. The motion of the pendulum mirror assembly is driven by the VCM, which consists of a coil fixed to the base and two magnets mounted on the pendulum mirror.

### 2.5. Other Devices

In addition to the FTIR spectrometer, the proposed gas detection system integrates supplementary devices including a pan-tilt unit, a visible light camera, an inertial navigation module (INM), and a micro industrial computer. These components collectively enhance the system’s practicality and reliability in real-world operating scenarios.

The pan-tilt unit features a maximum load capacity of 25 kg and supports horizontal and pitch motion. Equipped with an RS485 communication interface, its movement is controlled by serial port commands issued by the micro industrial computer. Detailed technical parameters of the pan-tilt unit are summarized in Table 3.

The visible light camera features a focal length of 100 mm, a resolution of 2048 × 2048 pixels, a pixel size of 11 μm × 11 μm, and a field of view of 30°. It transmits real-time images to the micro industrial computer via an Ethernet interface.

The inertial navigation module (INM) adopts the SBG system from France. Equipped with a built-in GPS receiver, the SBG system can acquire GPS data to provide positioning and motion parameters including latitude, longitude, altitude, and three-axis velocity. Its integrated gyroscope enables real-time feedback of three-dimensional angular information. The technical accuracy specifications of the output data are summarized in Table 4.

The micro industrial computer adopts the Dell 3000 series, configured with a 1 TB hard disk, multiple USB interfaces, an Ethernet interface, and a built-in wireless network card—effectively meeting the system’s data transmission and storage requirements. With dimensions of 182 mm × 178 mm × 36 mm and a total weight of only 650 g, the computer can be seamlessly integrated into the electronic control box, facilitating convenient installation.

### 2.6. Spectral Reconstruction and Gas Identification

**Figure 12:**
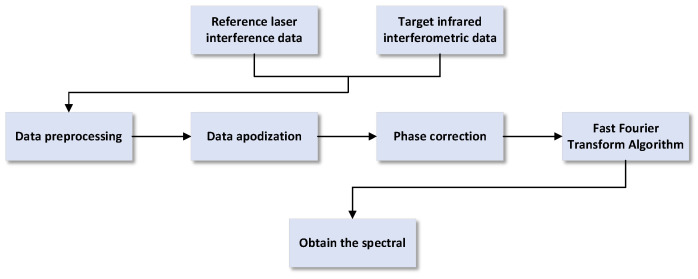
Spectral reconstruction workflow.

After the interferogram is acquired by the detector, spectral restoration via data processing techniques is required to obtain the final spectral data. The basic workflow of data processing is illustrated in the Figure 12.

**Figure 13:**
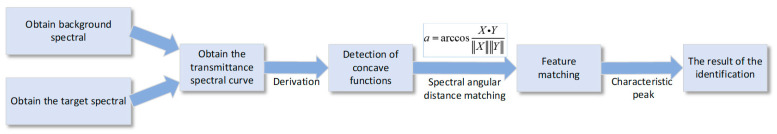
Substance identification workflow.

After acquiring the spectral data of both the measured target and the background, the infrared transmittance curve of the target is calculated. By comparing the information such as the position, range, and depth of characteristic peaks with the standard spectral curves, the identification of the target material composition is achieved. The specific workflow is shown in the Figure 13.

## 3. Results

### 3.1. Indoor Gas Detection Tests

The Noise Equivalent Column Concentration (NECL) is a critical indicator for the quantitative evaluation of gas telemetry systems. The expression for the system’s NECL is given as follows:

(2) $$NECL=\;-\frac{lg(1-\frac{NESR}{B\left(v\right)-L_{3}(v)})}{\sigma (v)}$$

The Noise Equivalent Spectral Radiance (NESR) refers to the standard deviation of the residual between the calibrated spectral radiance of the spectrometer and the theoretical spectral radiance within the target waveband range. Through calibration calculation, the NESR is determined to be 3.66 × 10^−8^ W/(sr·cm^2^·cm^−1^). *B* denotes the blackbody spectral radiance at the temperature of the target gas cluster, while *L*_3_ represents the blackbody spectral radiance at the background temperature, and *σ* is the absorption coefficient of the target gas. Sulfur hexafluoride (SF_6_) exhibits a distinct absorption peak at 947 cm^−1^, and its absorption coefficient obtained via simulation from the HITRAN database is approximately 3.1 × 10^−3^ m^2^/mg. When the gas cluster temperature is 25 °C and the background temperature is 35 °C, the calculated NECL of SF_6_ is 1.49 ppm·m.

**Figure 14:**
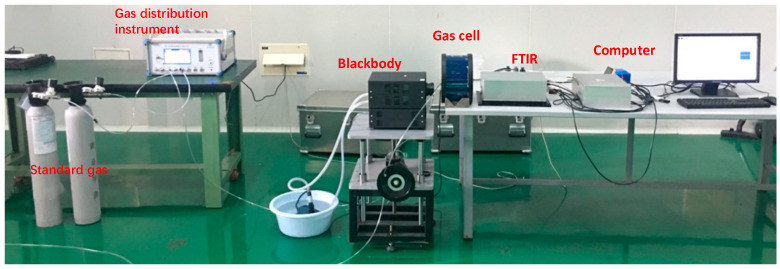
Schematic of the SF_6_ gas detection experimental setup.

To verify the sensitivity of the proposed gas detection system, FTIR-based spectral acquisition tests were conducted on SF_6_ gas samples with different concentrations in a laboratory environment. The experimental setup, illustrated in Figure 14, comprises a blackbody radiator, an automatic gas mixer, a gas cell, and standard SF_6_ gas cylinders. The blackbody radiator served as the background radiation source, while a gas cell with a 10 cm optical path was placed between the blackbody and the FTIR spectrometer.

**Figure 15:**
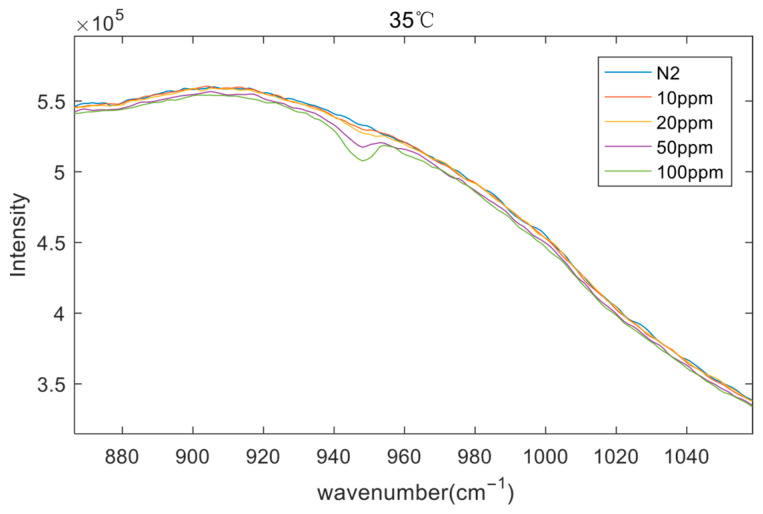
Raw spectra of SF_6_ gas at different concentrations (background temperature: 35 °C).

First, the gas cell is filled with nitrogen (N_2_, the concentration of SF_6_ is 0 ppm), and the infrared spectrum of N_2_ gas is collected. Then, an automatic gas mixer was used to prepare mixed gas samples of SF_6_ and N_2_ with SF_6_ concentrations of 100 ppm, 50 ppm, 20 ppm, and 10 ppm, respectively. These samples were sequentially injected into the gas cell, and their infrared spectra were collected. The experiments were performed under a laboratory temperature of 25 °C, with the background blackbody temperature set to 35 °C. Under this condition, the background temperature exceeded the gas temperature, resulting in distinct absorption peaks in the measured spectra of the target gas. As expected, the absorption intensity increased with the SF_6_ concentration, with higher concentrations exhibiting more prominent absorption features. The raw spectral data acquired from the tests are presented in Figure 15.

**Figure 16:**
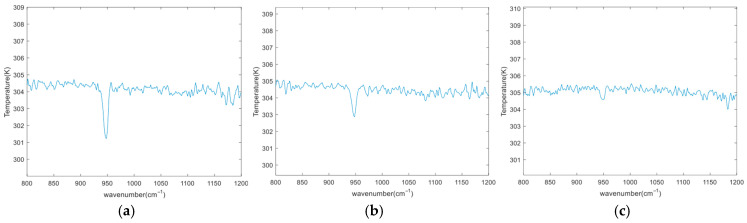
Brightness temperature spectra of SF_6_ gas at different concentrations (**a**) The concentration of SF_6_ is 100 ppm; (**b**) The concentration of SF_6_ is 50 ppm; (**c**) The concentration of SF_6_ is 20 ppm.

Subsequently, the raw spectra were calibrated via spectral radiation correction to extract the target gas’s brightness temperature spectra. Figure 16 depicts the brightness temperature spectra of SF_6_ gas at the tested concentrations (background temperature: 35 °C). The identification software is capable of accurately identifying SF_6_ gas at a concentration of 20 ppm.

**Figure 17:**
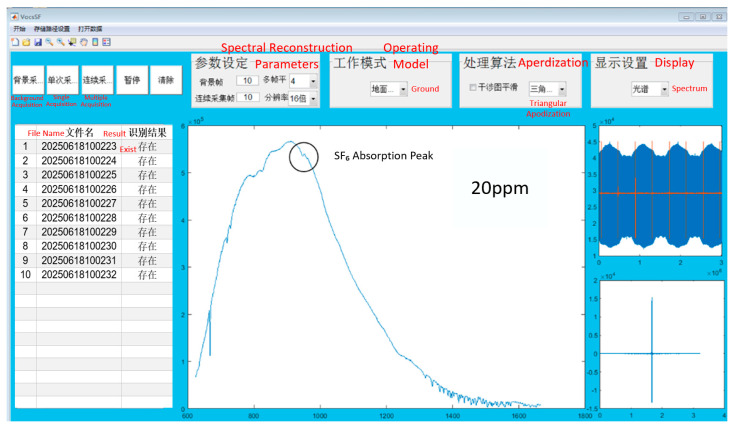
Statistics of multiple gas identification results.

After 10 measurements, statistical analysis was performed on the identification results, as shown in Figure 17. The detectable column concentration of SF_6_ gas by the FTIR system is calculated as 20 ppm × 10 cm = 2 ppm·m, which is basically consistent with the sensitivity obtained from the combination of theoretical analysis and simulation above.

### 3.2. UAV-Borne Gas Detection Field Test

To address the variable environmental conditions in outdoor field operations, the housing of the remote sensing FTIR spectrometer is sealed with rubber gaskets, and IP65-rated waterproof connectors are adopted to prevent moisture and dust from affecting the instrument. To mitigate the impact of drone vibrations on the spectrometer, shock absorbers are installed during its integration with the drone platform. Since strong winds can dilute the concentration of the target gas, the gas detection system operates in a hovering and staring mode during airborne detection to improve measurement accuracy.

A field test of the UAV-borne gas detection system was conducted in a bay area of Huizhou City, Guangdong Province. The ambient conditions at takeoff were a temperature of 20.8 °C and a wind speed of 4.8 m/s, with the UAV operating in aerial hovering mode during gas detection.

**Figure 18:**
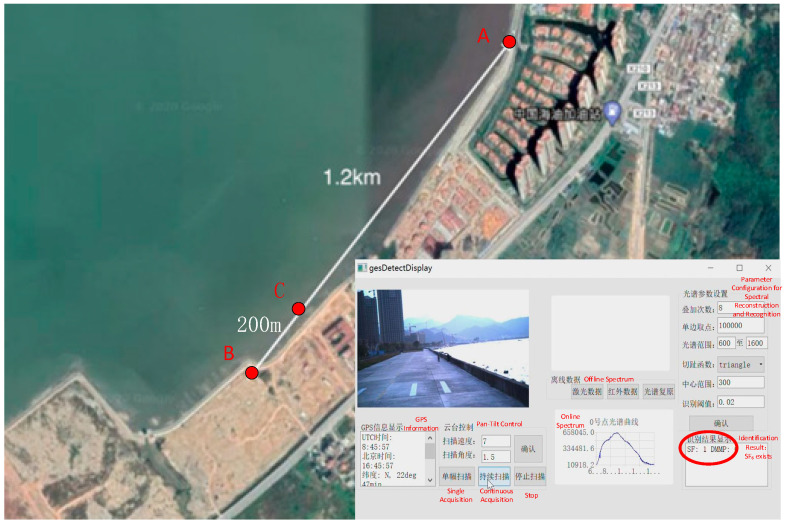
Flight test scenario and results.

As illustrated in Figure 18, Point A denotes the UAV’s takeoff location, Point B is the target gas release site, and Point C represents the UAV’s hovering position. The linear distance between Point A and Point B was 1.2 km. Upon flying to Point C—approximately 200 m away from the gas release point (Point B)—the UAV hovered and initiated continuous gas detection. The detection results of SF_6_ are shown in the Figure 18.

Since remote sensing FTIR relies on ambient thermal radiation as its light source, low ambient temperatures can compromise the accuracy of gas detection. In addition, because the absorption peaks of water vapor are densely distributed in the long-wave range, high water vapor content in the air can interfere with measurement accuracy. Therefore, the accuracy of gas detection can be improved to a certain extent in environments with relatively high temperatures and low humidity.

## 4. Conclusions

This paper presents the design, development, and validation of a lightweight, compact gas detection system based on passive FTIR spectroscopy. Characterized by its small form factor, operational flexibility, high optical throughput, superior spectral resolution, and rapid response capability, the system achieves high-sensitivity, real-time, and non-contact gas detection—addressing the critical need for comprehensive monitoring solutions in complex scenarios. To overcome the limitations of FTIR-based single-point measurement, the system is integrated with a visible light camera, a two-dimensional pan-tilt unit, and an INM. This multi-component integration enables precise localization of detection targets and expands the effective gas monitoring range, enhancing the system’s adaptability to practical applications.

In terms of technical optimization, significant innovations are implemented in the FTIR’s optical and electronic design: the optical core adopts an improved parallel pendulum mirror interferometer, which enhances structural stability and interference signal quality. To address the inherent challenge of weak infrared signal acquisition in passive FTIR spectroscopy, a low-noise signal preprocessing module with high signal-to-noise ratio is developed. Additionally, a hybrid data sampling method combining equal-time sampling and equal OPD sampling is employed, substantially improving the sampling accuracy of the data acquisition system and ensuring reliable spectral reconstruction.

Indoor experiments were conducted to detect SF_6_ gas samples with different concentrations. The detection limit of SF_6_ was ≤20 ppm under the conditions of a temperature difference of 10 °C and an optical path length of 10 cm, which verified the high sensitivity of the system. A flight test was carried out in the coastal area of Huizhou, Guangdong Province. Under complex operating conditions, the UAV hovered 200 m away from the target area for detection and successfully identified SF_6_ gas, which demonstrated the reliability and practicality of the system in real-world scenarios.

Compared with the passive FTIR spectrometers of Germany’s Bruker EM27 and America’s ABB OP-MR, which enjoy high international recognition, a comparison of their parameter indicators is presented in Table 5. The FTIR spectrometer designed in this paper features a more lightweight structure, along with high flexibility, high sensitivity and real-time non-contact detection capability. Its performance indicators can meet the demands for large-scale, high-precision gas detection in multiple fields such as pollution control, safety prevention and control, and environmental monitoring. Looking ahead, two key research directions will be prioritized for further development. First, a high-performance telescopic optical system will be developed to enhance the system’s long-range detection capability, enabling accurate gas identification at greater distances. Second, the system’s software algorithms will be optimized, with a focus on conducting systematic verification tests for multi-component mixed gas identification improving the system’s applicability to complex real-world scenarios. Thereby expanding its application scope to more challenging industrial, environmental, and emergency response scenarios.

## 5. Patents

One patents have been generated from this article, which is: A dual-channel orthogonal interferometer moving mirror control system. Patent No. ZL 2022 1 0647358.8

## Figures and Tables

**Table 1:** System parameter list.

Component	Parameter
Infrared spectrometer	1. Spectral region: 8–14 μm
	2. Spectral resolution: 1 cm^−1^
	3. Acquisition rate: 8 spectra/s
	4. Field angle: 1.5°
	5. Weight: <9 kg
	6. Size: 340 mm × 250 mm × 140 mm
Camera	1. Field angle: 30°
	2. Focal: 100 mm
Pan-Tilt Unit	1. Scan angel: −45~45°
	2. Scan speed: 0.01~20°/s

**Table 2:** Infrared detector parameter list.

Parameter	Value
D*	3.0 × 10^10^ cm·Hz^1/2^·W^−1^ (@1 kHz)
Spectral Region	2~14 μm
Responsivity	3000 V/W@24.5 mA
Element Size	1 mm × 1 mm
Operating Temperature	77 K
Field of View	60°

**Table 3:** Pan-tilt unit parameter list.

Parameter	Value
Horizontal Angle Range/Speed	±45°/0.01~20°/s
Pitch Angle Range/Speed	±15°/0.01~10°/s
Positional Accuracy	0.1°
Maximum Load	25 kg
Communication Interface	RS-485
Voltage/Power	28 VDC/<70 W

**Table 4:** Inertial navigation module parameter list.

Parameter	Accuracy
Roll angle/Pitch angle	0.1°
Azimuth	<0.5°
Speed	0.1 m/s
Position	2 m
Acceleration range	±16 g
Barometric pressure accuracy	1.2 Pa

**Table 5:** Comparison of commercial spectrometer parameters.

Parameter	ABB OP-MR	Bruker EM27	Present Design
Spectral region	1.5–13.5 μm	2–14 μm	8–14 μm
Spectral resolution	1 cm^−1^	1 cm^−1^	1 cm^−1^
Acquisition rate	2 spectra/s	5 spectra/s	8 spectra/s
Field angle	1.43°	1.7°	1.5°
Weight	35 kg	18 kg	9 kg
Size	587 mm × 273 mm× 372 mm	400 mm × 360 mm× 270 mm	340 mm × 250 mm × 140 mm

## Data Availability

The original contributions presented in this study are included in the article. Further inquiries can be directed to the corresponding author.

## Abbreviations

The following abbreviations are used in this manuscript:

FTIR	Fourier Transform Infrared Spectroscopy
TDLAS	Tunable Diode Laser Absorption Spectroscopy
GC-MS	Gas Chromatograph-Mass Spectrometer
SNR	Signal-to-Noise Ratio
INM	Inertial Navigation Module
MCT	Mercury Cadmium Telluride
VCM	Voice Coil Motor
UAV	Unmanned Aerial Vehicle
BS	Beam Splitter
OPD	Optical Path Difference
GPS	Global Position System
NECL	Noise Equivalent Column Concentration
NESR	Noise Equivalent Spectral Radiance

## References

[1] Tian H., Wu Z. (2022). Electrical Resistance Sensor Based on ZnO Nanoarray for VOC Gas Detection. Int. J. Electrochem. Sci..

[2] Zhang R.-X., Zong X.-H., Yu T.-T., Ge Y.-X., Hu S., Liang W.-J. (2022). Detection and identification of gas components based on nano sensor array. Acta Phys. Sin..

[3] Schmithausen A.J., Trimborn M., Büscher W. (2016). Methodological Comparison between a Novel Automatic Sampling System for Gas Chromatography versus Photoacoustic Spectroscopy for Measuring Greenhouse Gas Emissions under Field Conditions. Sensors.

[4] Wang B., Tang X., Gan Y., Li X., Lu Y. (2022). A TC/WMS-TDLAS Mid-Infrared Detection Method for Ultra-Low Concentration Carbon Isotope Methane. J. Anal. At. Spectrom..

[5] Andreev M., Platonov V., Filatova D., Galitskaya E., Polomoshnov S., Generalov S., Nikolaeva A., Amelichev V., Zhdaneev O., Krivetskiy V. (2021). Flame-Made La_2_O_3_-Based Nanocomposite CO_2_ Sensors as Perspective Part of GHG Monitoring System. Sensors.

[6] Thériault J.M., Puckrin E., Lavoie H. (2007). Remote Monitoring of Multi-Gas Mixtures by Passive Standoff Fourier Transform Infrared Radiometry. Appl. Spectrosc..

[7] Ben-David A., Ren H. (2003). Detection, identification, and estimation of biological aerosols and vapors with a Fourier-transform infrared spectrometer. Appl. Opt..

[8] Lavoie H., Bouffard F., Puckrin E., Thériault J. (2021). Standoff detection of hazardous gas in open environments, two decades of R&D activities at DRDC. Proceedings of the OSA Optical Sensors and Sensing Congress 2021, Washington, DC, USA, 19–23 July 2021.

[9] Qin Y., Tong J., Li X., Han X., Gao M. (2023). The Effect of Spectral Resolution on the Quantification of OP-FTIR Spectroscopy. Photonics.

[10] Schütze C., Sauer U. (2016). Challenges associated with the atmospheric monitoring of areal emission sources and the need for optical remote sensing techniques—An open-path Fourier transform infrared (OP-FTIR) spectroscopy experience report. Environ. Earth Sci..

[11] Augustyniak D., Szala M. (2025). Field Explosives Detectors—Current Status and Development Prospects. Sensors.

[12] Jang H.-D., Kwon S., Nam H., Chang D.E. (2024). Semi-Supervised Autoencoder for Chemical Gas Classification with FTIR Spectrum. Sensors.

[13] Zhang F., Zhu Y., Li L., Zhao S., Zhang X., Chen C. (2025). Research on Quantitative Analysis Method of Infrared Spectroscopy for Coal Mine Gases. Molecules.

[14] Friedl-Vallon F., Maucher G., Seefeldner M., Trieschmann O., Kleinert A., Lengel A., Keim C., Oelhaf H., Fischer H. (2004). Design and characterization of the balloon-borne Michelson Interferometer for Passive Atmospheric Sounding (MIPAS-B2). Appl. Opt..

[15] Xu L., Liu J., Liu W., Jin L., Gao M., Hu R., Ye S., Li Y., Hu Y. (2016). Industrial Air Pollution Monitoring by Active and Passive Fourier Transform Infrared Spectroscopy. Proceedings of the Light, Energy and the Environment 2016, Leipzig, Germany, 14–17 November 2016.

[16] Schulenburg N.W. (2002). Real-time processing for remote gas identification. Proceedings of the SPIE 4745, Systems, and Architectures for Trans-National Defense, Orlando, FL, USA, 1–5 April 2002.

[17] Lu X., Huang M., Han W., Qian L., Wang Z., Sun Y. (2024). Sampling Error Analysis of FTIR and Design of Low Noise Sampling System. Proceedings of the 2024 IEEE ICSPCC, Bali, Indonesia, 19–22 August 2024.

[18] Bu H., Wei H., Wu S., Wang G. (2020). Real-time analysis and simulation of remote sensing photoelectric information from aerospace Fourier transform infrared spectrometer. Proceedings of the SPIE 11566, AOPC 2020: Optical Spectroscopy and Imaging, and Biomedical Optics, Beijing, China, 30 November–2 December 2020.

[19] Bekker D.L., Blavier J.-F.L., Toon G.C., Servais C. (2009). An FPGA-based data acquisition and processing system for the MATMOS FTIR instrument. Proceedings of the 2009 IEEE Aerospace Conference, Big Sky, MT, USA, 7–14 March 2009.

[20] Pougatchev N.S., Campbell J.F., Regan C.R., Abrams M.C., Brault J.W., Farmer C.B., Hinton D.E. (2000). Advanced technologies high resolution Fourier transform spectrometer for atmospheric studies. Proceedings of the 2000 IEEE Aerospace Conference, Big Sky, MT, USA, 25 March 2000.

[21] Tonnisson T. (2008). Compact Fourier transform infrared spectrometer module. Proceedings of the 2008 11th International Biennial Baltic Electronics Conference, Tallinn, Estonia, 6–8 October 2008.

